# Assessment of cardiopulmonary manifestations and its correlation with semi-quantitative scoring of high-resolution computed tomography in patients with autoimmune rheumatic diseases

**DOI:** 10.1186/s12890-023-02404-9

**Published:** 2023-04-19

**Authors:** Mai M. El-Kalashy, Samah A. Elbeltagy, Enas S. Zahran, Maha M. Salman, Shrief R. Abd Elrahman, Mai M. Abdalraouf, Amal A. El-Koa

**Affiliations:** 1 Chest Disease and Tuberculosis, Faculty of Medicine, Menoufia University, Shibin Al Kawm, Egypt; 2 Internal Medicine, Rheumatology and Clinical Immunology Unit, Faculty of Medicine, Menoufia University, Shibin Al Kawm, Egypt; 3 Physical Medicine, Rheumatology and Rehabilitation, Faculty of Medicine, Menoufia University, Shibin Al Kawm, Egypt; 4 Radiodiagnosis, Faculty of Medicine, Menoufia University, Shibin Al Kawm, Egypt; 5 Cardiology, Faculty of Medicine, Menoufia University, Shibin Al Kawm, Egypt

**Keywords:** HRCT, Autoimmune rheumatic diseases, Semi-quantitative score, Echocardiography

## Abstract

**Purpose:**

Autoimmune rheumatic diseases (ARD) are groups of diseases that are commonly associated with cardiac and pulmonary manifestations and may affect the morbidity and mortality of the patients. The study aimed to the assessment of cardiopulmonary manifestations and their correlation with the semi-quantitative scoring of high-resolution computed tomography (HRCT) in ARD patients.

**Methods and patients:**

30 patients with ARD were included in the study (mean age 42.2 ± 9.76 years) [10 patients were scleroderma (SSc), 10 patients were rheumatoid arthritis (RA), and 10 patients were systemic lupus erythematosus (SLE)]. They all met the diagnostic criteria of the American College of Rheumatology and underwent spirometry, echocardiography, and chest HRCT. The HRCT was assessed by a semi-quantitative score for parenchymal abnormalities. Correlation between HRCT lung scores and: inflammatory markers, lung volumes in spirometry, and echocardiographic indices has been performed.

**Results:**

The total lung score (TLS) by HRCT was 14.8 ± 8.78 (mean ± SD), ground glass opacity score (GGO) was 7.20 ± 5.79 (mean ± SD) and fibrosis lung score (F) was 7.63 ± 6.05 (mean ± SD). TLS correlated significantly with ESR (r 0.528, p 0.003), CRP (r 0.439, p 0.015), PaO2 (r -0.395, P 0.031) FVC% (r -0.687, p 0.001), and echocardiographic Tricuspid E (r -0.370, p 0.044), Tricuspid E/è (r -0.397,p 0.03), ESPAP (r 0.459,p 0.011), TAPSE (r -0.405, p 0.027), MPI-TDI (r -0.428, p 0.018) and RV Global strain(r -0.567, p 0.001). GGO score correlated significantly with ESR (r 0.597, p 0.001), CRP (r 0.473, p 0.008), FVC% (r -0.558, p 0.001), and RV Global strain(r -0.496, p 0.005). F score correlated significantly with FVC% (r -0.397, p 0.030), Tricuspid E/è (r -0.445, p 0.014), ESPAP (r 0.402, p 0.028), and MPI-TDI (r -0.448, p 0.013).

**Conclusion:**

The total lung score and GGO score in ARD were found to be consistently significantly correlated with FVC% predicted, PaO2, inflammatory markers, and RV functions. Fibrotic score correlated with ESPAP. Therefore, in a clinical setting, most clinicians who monitor patients suffering from ARD should concern with the applicability of semiquantitative HRCT scoring in clinical practice.

## Introduction

Autoimmune rheumatic diseases (ARD) are disorders with autoimmune characteristics that can influence any of the body systems causing organ damage. Examples include scleroderma or progressive systemic sclerosis (SSc), rheumatoid arthritis (RA), autoimmune myopathies, and systemic lupus erythematosus (SLE) [1]. They can involve any part of the cardiac or pulmonary system and are commonly accompanied by interstitial lung diseases (ILDs) which might be the primary cause of death in most patients, especially with usual interstitial pneumonia [2, 3].

Rather than being dependent on definite validation, the utility of the HRCT in the identification of ILDs indicates universal practical knowledge of discontent with alternative methodologies. In other words, during the last 20 years, HRCTs is practically beneficial in the identification of ILDs; as a result, they acquired a crucial role [4].

HRCT has been considered the gold standard in the detection of ARD-related ILD, particularly in the early stages of the disease. [5, 6]. The most prevalent radiologic feature in ARD is diffuse parenchymal interstitial lung disease (ILD), which can manifest by ground glass opacity (GGO), reticulation, tractional bronchiectasis, or honeycombings (HC) [7].

A lot of CT scoring system was described in the ILD associated with ARD, some automated or computer-aided, and others depend on reader visual assessment and semiquantitative (semi-QA) methods [8, 9].

Although many studies looked at the degree and severity of ILD detected by HRCT and whether they were correlated with the level of respiratory insufficiency and lung functions, the majority of studies focused on SSc patients rather than all ARD patients, and only a small number of studies linked CT scoring to right ventricular function [10–13]. More knowledge concerning the link between distinct CT abnormalities and underlying disease pathophysiology and illness progression may be gained by assessing the relation of any abnormalities with the clinical data [14]. So, in this study, the spirometry, inflammatory markers including ESR and CRP, and right ventricular (RV) function by echocardiography were assessed, together with their correlation with semi-quantitative scoring of HRCT in the patients with different ARD.

## Patients and methods

### Study population

This prospective study was conducted on 30 patients (mean age 42.2 ± 9.76 years), diagnosed with ARD, attending Menoufia University Hospitals from the period of January 2021 to March 2022. All of the patients met the diagnostic criteria of the American College of Rheumatology that including RA [15], SSc [16], and SLE [17], and their age was > 18 years old. Exclusion criteria were any patient with chronic pulmonary, or cardiac disease, lung cancer history, a known cause of interstitial lung fibrosis e.g., occupational, age less than 18 years old, patients, who couldn’t perform spirometry, or patient refusal. The Menoufia University Hospital Research Ethical Committee gave its approval to the study (IRB: 6/2022CHES4-2).

### Study design

The patients underwent comprehensive history taking, clinical evaluations, and basic laboratory tests like C-reactive protein (CRP) and erythrocyte sedimentation rate (ESR) and PaO_2_(mmHg), spirometry, HRCT, and echocardiography.

### Spirometric function evaluation

Spirometry was done using a computerized spirometer (THOR Laboratories Kft Spirometer, Hungary) in the Unit of pulmonary function test (PFT) in Menoufia University Hospital, taking the data with a particular emphasis on the ratio of forced expiratory volume in 1st second (FEV1) / forced vital capacity (FVC) and FVC% of the predicted values following the guidelines of the American Thoracic Society/European Respiratory Society (ATS/ERS) [18]. The test was repeated at least 2 times to report the best values. Restrictive abnormality was determined if the FVC% < 80% together with a normal ratio of FEV1/FVC.

### Transthoracic echocardiography (ECHO)

The ECHO was performed with an S4-2 probe (Vivid9, General Electric health care Vingmed, Norway) by an experienced echocardiographer and all patients underwent:

1. **Conventional 2-dimensional (2D) transthoracic ECHO** with functional evaluation of the RV which includes:[19] measuring EPASP (using the tricuspid regurgitation velocity and the Bernoulli equation), Tricuspid annular plane systolic excursion (TAPSE), RV-fractional area change (RV-FAC), TDI derived RV myocardial performance index (TDI-MPI), pulsed wave derived tricuspid E wave and A wave and early tricuspid diastolic wave velocity (è) and E/ è ratio that measured by TDI to assess the diastolic function of the RV.
2. **The 2 D-speckle tracking echocardiography (2D STE)**: to assess and analyze the RV global strain as the conventional 2-D ECHO isn’t sensitive enough for detection of subclinical RV changes in autoimmune disease.

### HRCT assessment and HRCT score

All patients underwent a multi-slice CT study without contrast for the chest. The CT was performed using multi-slice 16 detectors (CT Toshiba Aquilion 1) and the CT acquisition parameters were as follows: Slice thickness was set at 1.0 mm, reconstruction interval was between 1.0 and 3.0 mm, and a sharp reconstruction technique was employed. The tube voltage ranged from 120 to 160 kVp.

### Semiquantitative (SemiQA) scoring of HRCT

**Fig. 1:**
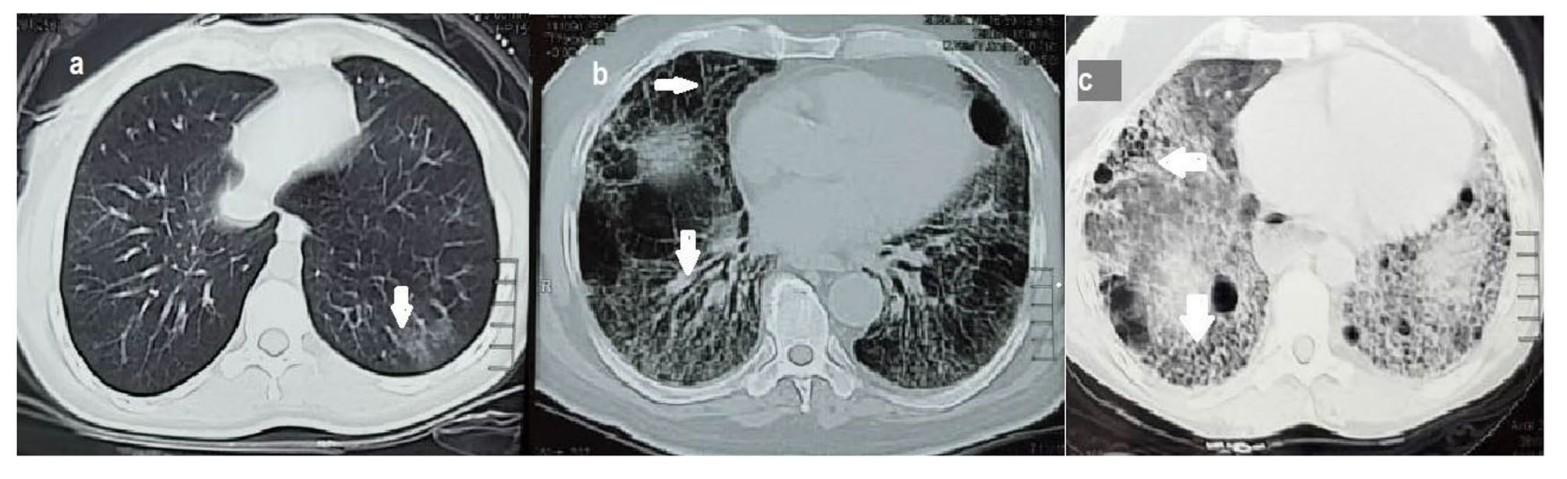
shows the parynchymal abnormalities in HRCT from different patients. (a): pure GGO, (b): reticulations and traction bronchiectasis, and (c): honeycombing changes

Semi-quantitative scoring is determined by evaluating the extent of the illness and assigning a score; higher scores coincide with more progression of the disease. All CTs were examined by an expert chest radiologist blinded to any of the clinical presentations or the diagnosis of the patient. We used the scoring methods modified from that described by **Goldin et al.** [20] used in the Scleroderma Lung Study (Table 1). In this study, The upper, middle, and lower zones of each lung were divided, and each zone was independently scored. From the lung apex to the arch of the aorta is considered the upper zone, the middle zone is from the arch of the aorta to the inferior pulmonary veins, while from the inferior pulmonary veins to the diaphragm is considered the lower zone. The abnormalities were divided into ground-glass opacity (GGO) and total fibrosis (F) (Fig. 1). GGO is defined radiologically as a hazy opacity in the lung parenchyma without any reticulations or architectural distortion and F is defined as any reticulations (inter- or intra-lobular), traction bronchiectasis, or honeycombing (HC) which is defined as multiple layers of dense walled air-filled cysts [21]. The grading of abnormality extension is expressed in a scale in which (0 = absent; 1 = 1–25%; 2 = 26–50%; 3 = 51–75% and 4 = 76–100%). Finally, The total CT score was defined as the summation of the grades in the Six zones. This scoring was derived from the scoring of **Kazerooni et al.** [22] in which the lung zones were changed into lobes.

### Statistical analysis

An IBM personal computer running version 20 of the Statistical Package of Social Science was used to analyze the data (SPSS, Inc, Chicago, Illinois, USA). The qualitative data were presented as % and numbers, while the quantitative data were presented as mean, standard deviation (SD), and range. Quantitative data were correlated using Spearman’s correlation. P values lower than 0.05 were deemed statistically significant.

## Results

This study was conducted on 30 patients (mean age 42.2 ± 9.76 years, 27 females and 3 males) diagnosed with ARD; 10 patients with SSc, 10 patients with RA, and 10 patients with SLE. The duration of the disease ranged between 2 and 15 years. The Socio-demographic together with the clinical data of the studied participants shown in (Table 2).

Table 3 showed mean values of HRCT scoring, laboratory parameters, spirometry values, and ECHO finding among the studied patients.

The total lung score was 14.8 ± 8.78 (mean ± SD) where GGO was 7.20 ± 5.79, and the total fibrosis (F) was 7.63 ± 6.05.

Correlations between total lung score, GGO score, and lung fibrosis score by HRCT and: inflammatory markers, echocardiographic indices as well as lung volumes by spirometry are presented in Table 4.

Total lung score showed statistically significant positive correlations with inflammatory markers (ESR and CRP (r 0.528, p 0.003 and r 0.439, p 0.015 respectively) together with ESPAP (r 0.459, p 0.011). Furthermore, it was statistically significant and inversely correlated with PaO_2_(r -0.395, P 0.031), FVC% (r -0.687, p 0.001), and RV functions including Tricuspid E(r= -0.370, p = 0.044), Tricuspid E/è (r -0.397,p 0.03), TAPSE (r -0.405, p 0.027), MPI-TDI(r -0.428, p 0.018) and RV Global strain (r -0.567, p 0.001).

GGO score correlated significantly with ESR (r 0.597, p 0.001), CRP (r 0.473, p 0.008), FVC% (r -0.558, p 0.001), and RV Global strain(r -0.496, p 0.005).

F score correlated significantly with FVC% (r -0.397, p 0.030), Tricuspid E/è (r -0.445,p 0.014), ESPAP (r 0.402,p 0.028), and MPI-TDI (r -0.448, p 0.013).

## Discussion

HRCT is considered a noninvasive reference approach for the diagnosis of ILD because it gives good parenchymal detail [23, 24].

Semi-quantitative scoring is determined by evaluating the extent of the illness and assigning a score; higher scores coincide with more progression of the disease, while the exact percent of lung involvement is employed in quantitative approaches [11]. In this current study, we use the semi-QA scoring method described by **Goldin et al.** [20], the method described in the Scleroderma Lung Study. The main results of this study were that spirometric FVC%, PaO2, inflammatory markers, and RV functions by ECHO were correlated with the total lung score by the Semi-QS method in ARD patients.

Semi-QA scoring methods, in multiple studies, were compared to QA using computer-aid approaches. Computer-aid methods were well correlated with the visual scoring methods in the identification of lung fibrosis together with the assessment of the disease extent and there was no intrareader variation encountered in visual scoring methods [8, 25–28]. So, we tried to use the Semi-QA scoring in HRCT in patients with ARD and its correlation with the inflammatory markers, spirometry, and echocardiographic RV functions.

In this current study, the total lung score was 14.8 ± 8.78 (mean ± SD) where GGO was 7.20 ± 5.79 and the total fibrosis (F) was 7.63 ± 6.05. The spirometry showed restrictive type with the FVC % − 64.4 ± 9.20 (mean ± SD) and FEV1/FVC% − 82.1 ± 6.03 A significant inverse correlation between the total lung score, GGO score, and F score with the FVC % was found (r -0.687, p 0.001; r -0.558, p 0.001 and r -0.397, p0.030 respectively).

The spirometry in our study coincided with the study of **Mena-Vázquez et al.** that showed the character and progress of ILD in ARD patients between the period of 2015 and 2020. The FVC of all patients (n = 204) was 72 ± 16.6 (mean ± SD) and at the end of the study was 68.2 ± 16.2(mean ± SD) [29].

In the study of **Marten et al.**, all patients with collagen vascular disease (CVD) (n = 52) had findings of ILD in thin-section CT. The extent of ILD was 36.3 ± 27.2% determined by the readers (the extent of reticulation was 27.0 ± 23.3%, the GGO extent was 9.2 ± 17.0%, the extent of the coarseness of the reticulation was 1.1 ± 0.6%). There were significant correlations between the ILD extent in CT and FVC and FEV1 (r − 0.559, P 0.0002 and r = − 0.379, P = 0.014 respectively). The reticulation extent was correlated moderately with FVC (r = − 0.436, P = 0.005), while no significant correlations were found between FEV1 and reticulation extent. There were no significant correlations between the GGO, and coarseness extent with either FVC or FEV1 [30].

Most studies used the semi-quantitative scoring systems to assess the PFT in Systemic sclerosis patients and found that the total score of HRCT was significantly and negatively correlated with TLC, FVC%, and DLCO [10, 31–33].

Our results were near that of **Zexuan et al.** who involved the scleroderma patients using the scoring system used by Goldin et al., they found that the total score was 14.35 ± 6.18 where GGO was 7.15 ± 3.94, PF 5.58 ± 2.91, and HC 1.36 ± 2.66. But they put also emphysema in the scoring which was 0.28 ± 1.05 [34].

**Goldin et al.** studied the HRCT scan on 162 scleroderma patients. They found that the F and the GGO scores were the commonest abnormalities in symptomatic scleroderma patients, and the extent of the F score had a statistically significant negative correlation with DLCO (r − 0.44), FVC (r − 0.22), and TLC (r − 0.36). They suggested that pure GGO on CT might be reversible as it represents inflammation because its extent was correlated with acute inflammatory cells in bronchoalveolar fluid and not correlated with PFT (r = 0.28) [20].

The study of **Wangkaew et al.** involved 31 participants with SSc and used the HRCT score that classified the parenchymal abnormalities into 4 items: GGOs, lung fibrosis, bronchiectasis, and HCs. In concordance with our study, they found a significant and inverse correlation between FVC % and total GGO (r − 0.43; P 0.05), total Fibrosis score (r − 0.56; P 0.01), total bronchiectasis score (r − 0.43; P 0.05) and total scores of HRCT (r − 0.52; P 0.01) together with significant correlations between HRCT scores and O2 saturation (r − 0.47; P 0.01) and ESR (r 0.38, P 0.05) [35].

We tried to assess the correlation of total lung score with right ventricular echocardiographic function in different ARD. We found that the total lung score has statistically significant correlations with Tricuspid E (r -0.370, p 0.044), Tricuspid E/è (r -0.397, p 0.03), TAPSE (r -0.405, p 0.027), MPI-TDI (r -0.428, p 0.018), RV Global strain (r -0.567, p 0.001), and ESPAP (r 0.459, p 0.011). The total F score was significantly correlated with EPASP (r 0.402, p 0.028), Tricuspid E/è (r -0.445, p 0.014), and MPI-TDI (r -0.448, p 0.013).

There were limited studies that correlate the CT score in ARD with RV function by ECHO. For example, **Pandey et al.** found that the total HRCT score correlated with elevated pulmonary arterial pressures (PAP). Their study showed a significant relationship between the peak PAP and the total CT score (p < 0.0001). They stated that the fibrotic score was the most determinant factor of pulmonary hypertension (PH) on ECHO, which can help in the screening of SSc patients for PH by the extent of pulmonary fibrosis [13].

Some patients had significant elevations in the PAP although they had small or no lung fibrosis. This could explain why multiple factors contribute to the occurrence of PH in people with scleroderma, including pulmonary vasculopathy and capillary bed obliteration, and lung fibrosis may be the main factor [13].

The study by **Mukerjee et al.** found that the EPASP on echo (mmHg) was 39 ± 15 in scleroderma patients without lung fibrosis and 46 ± 18 in scleroderma patients with lung fibrosis detected by HRCT [36]. In the study of **Mohammed et al.**, the mean EPASP in the studied SLE patients was 31 ± 5.1 mmHg with no significant variation between the diseased and control group [37].

There were some limitations in this study e.g., the total number of participants was small in each group of diseases, not all diseases of ARD are involved, the scoring system used didn’t include other CT abnormalities e.g., emphysematous changes in ARD, and more PFT parameters need to be evaluated e.g., TLCO. We recommend further studies on a large number of participants with ARD with the comparison between different types of CT scoring systems that include more abnormalities recording.

## Conclusion

Semi-quantitative approaches of HRCT are distinguished by the precise estimation of interstitial lung disease extent and character. The higher grade equates to more advanced lung disease. The total lung score and GGO score in ARD were found to be consistently significantly correlated with FVC% predicted, PaO2, inflammatory markers, and RV functions. Fibrotic score correlated with ESPAP. Therefore, in a clinical setting, most clinicians who monitor patients suffering from ARD should concern with semi-quantitative HRCT scoring in clinical practice.

**Table 1:** The Socio-demographic and the clinical data of the studied patients (N = 30)

Studied variables	Studied patients (N = 30)
Age/years Mean ± SD Median Range	42.2 ± 9.76 38.5 27.0–61.0
Sex Male Female	7(23.3) 23(76.7)
**Smoking** Yes No	3(10.0) 27(90.0)
**DM** Yes No	10(33.3) 20(66.67)
**Hypertension** Yes No	5(16.7) 25(83.3)
**Cough** Yes No	27(90.0) 3(10.0)
**Dyspnea** Grade II Grade III	6(20.0) 24(80.0)
**Fever** Yes No	3(10.0) 27(90.0)
**Lower limb Edema** Yes No	7(23.3) 23(76.7)
**Crepitation** Yes No	29(96.7) 1(3.30)
**Wheezes** Yes No	3(10.0) 27(90.0)

**Table 2:** Laboratory investigations, spirometry, HRCT scores, and ECHO findings among the studied patients (N = 30)

Studied variables	Mean ± SD	Min - Max
**ESR**	42.7 ± 12.9	24.0–66.0
**CRP**	14.9 ± 13.1	0.60–51.0
**PO2**	69.8 ± 6.08	55.0–80.0
**FVC**	64.4 ± 9.20	41.0–78.0
**FEV1/FVC**	82.1 ± 6.03	70.0–100
**HRCT scores**		
**Total lung score**	14.8 ± 8.78	1.00–33.0
**GGO score**	7.20 ± 5.79	0.00–20.0
**Fibrosis score**	7.63 ± 6.05	0.00–18.0
**ECHO findings**		
**Tricuspid E**	0.58 ± 0.17	0.26–1.15
**Tricuspid A**	0.72 ± 0.23	0.31–1.22
**Tricuspid è**	0.12 ± 0.02	0.07–0.18
**Tricuspid E/è**	4.86 ± 2.39	2.74–14.3
**EPASP**	38.5 ± 9.25	22.0–56.0
**TAPSE**	1.80 ± 0.45	1.10–2.60
**RVFAC**	43.9 ± 10.9	29.0–81.0
**MPI-TDI**	0.71 ± 0.16	0.46–1.09
**RV Global strain**	-16.3 ± 3.87	-9.10 – -25.7

**Table 3:** Correlation between HRCT scores and Laboratory investigations, spirometry, and ECHO findings among the studied patients (N = 30)

Studied variables	Total lung score		GGO score		Fibrosis score	
	r	P	r	P	r	P
**ESR**	0.528	0.003**	0.597	0.001**	0.200	0.290
**CRP**	0.439	0.015*	0.473	0.008**	0.180	0.342
**PO2**	-0.395	0.031*	-0.320	0.085	-0.243	0.196
**FVC**	-0.687	0.001**	-0.558	0.001**	-0.397	0.030*
**FEV1/FVC**	0.051	0.789	0.120	0.526	0.067	0.724
**Tricuspid E**	-0.370	0.044*	-0.176	0.353	-0.285	0.126
**Tricuspid A**	-0.113	0.552	0.170	0.368	-0.269	0.151
**Tricuspid è**	0.251	0.180	0.094	0.620	0.319	0.086
**Tricuspid E/è**	-0.397	0.030*	-0.076	0.689	-0.445	0.014*
**EPASP**	0.459	0.011*	0.205	0.277	0.402	0.028*
**TAPSE**	-0.405	0.027*	-0.279	0.136	-0.354	0.055
**RVFAC**	-0.259	0.166	-0.050	0.792	-0.343	0.064
**MPI-TDI**	-0.428	0.018*	-0.135	0.477	-0.448	0.013*
**RV Global strain**	-0.567	0.001**	-0.496	0.005**	0.154	0.416

*P < 0.05, **P < 0.01

**Table 4:** Semi-quantitative scoring modified from that used in Scleroderma Lung Study by Goldin et al. [20]

Parenchymal abnormalities	Grading abnormality		Anatomical regions that scored
	Percentage of disease extent	score	
-Pure ground-glass opacity -Total Fibrosis (including thickened reticular markings, bronchiectasis, and Honeycombing)	0 1–25% 26–50% 51–75% > 75%	0 1 2 3 4	Zone 1: Apex to Aortic arch Zone 2: Aortic arch to inferior pulmonary veins Zone 3: Inferior pulmonary veins to diaphragms Right and left lungs scored separately

## Data Availability

On reasonable request, the corresponding author will provide the datasets used and/or analyzed during the current work.

## List of abbreviations

**ARD**: Autoimmune rheumatic diseases
**CRP**: C-reactive protein
**ECHO**: Echocardiography
**EPASP**: Estimated pulmonary artery systolic pressure
**ESR**: Erythrocyte sedimentation rate
**F**: Fibrosis
**FEV1/ FVC**: forced expiratory volume in 1st second / forced vital capacity
**GGO**: Ground glass opacity
**HC**: Honeycombing
**HRCT**: high-resolution computed tomography
**ILD**: interstitial lung disease
**MPI**: myocardial performance index
**PFT**: Pulmonary function test
**RA**: rheumatoid arthritis
**RV-FAC**: right ventricle- fractional area change
**semi-QA**: semiquantitative
**SLE**: systemic lupus erythematosus
**SSc**: scleroderma
**STE**: speckle tracking echocardiography
**TAPSE**: Tricuspid annular plane systolic excursion
