# Correction: Assessment of cardiopulmonary manifestations and its correlation with semi-quantitative scoring of high-resolution computed tomography in patients with autoimmune rheumatic diseases

**DOI:** 10.1186/s12890-024-03111-9

**Published:** 2024-06-25

**Authors:** Mai M. El-Kalashy, Samah A. Elbeltagy, Enas S. Zahran, Maha M. Salman, Shrief R. Abd Elrahman, Mai M. Abdalraouf, Amal A. El-Koa

**Affiliations:** 1 Chest Disease and Tuberculosis, Faculty of Medicine, Menoufia University, Shibin Al Kawm, Egypt; 2 Internal Medicine, Rheumatology and Clinical Immunology Unit, Faculty of Medicine, Menoufia University, Shibin Al Kawm, Egypt; 3 Physical Medicine, Rheumatology and Rehabilitation, Faculty of Medicine, Menoufia University, Shibin Al Kawm, Egypt; 4 Radiodiagnosis, Faculty of Medicine, Menoufia University, Shibin Al Kawm, Egypt; 5 Cardiology, Faculty of Medicine, Menoufia University, Shibin Al Kawm, Egypt

**Correction: BMC Pulmonary Medicine (2023) 23:131**

10.1186/s12890-023-02404-9

Following publication of the original article, it came to the authors’ attention that the article had published with an error in the tables: the captions of the tables did not correctly match the tables. The captions of the tables have since been corrected in the published article. This correction does not affect the order of the tables themselves nor any of the references to the tables in the article text.

The authors thank you for reading this erratum and apologize for any inconvenience caused.

